# The origins, characteristics and trends of neo-nationalism in the 21st century

**DOI:** 10.1186/s41257-022-00079-4

**Published:** 2022-12-08

**Authors:** Shaoqing Zhou

**Affiliations:** Institute of Ethnology and Anthropology Chinese Academy of Social Sciences, Beijing, China

**Keywords:** Neo-nationalism, Evangelical nationalism, Far-right nationalism, Separatist nationalism, Third world religious nationalism

## Abstract

The rise of neo-nationalism has been an important political phenomenon since the 21st century. Neo-nationalism is not a single form of nationalism. It is not only a generalization of a specific type of nationalism at present, but also a description of a series of new nationalism phenomena. From the point of what it may include, it has at least four forms: far-right nationalism, evangelical nationalism, separatist nationalism, and (the third world) religious nationalism. Compared with traditional nationalism, neo-nationalism has undergone major changes in terms of guiding ideology or values behind, epochal character, propulsion mechanism, function, propagation mode, influence, and field of occurrence. At the same time, neo-nationalism is also a kind of high-intensity identity politics with a sort of quasi-fundamentalist characteristics. It discards the core value principles of traditional nationalism and the basic etiquette of polite society. Some dangerous and even crazy essential factors of nationalism have been developed to extremes in the new era. This means that its destructiveness to specific countries and societies is far greater than that caused by the reactive identity politics of minorities. From the perspective of trends in development, while the neo-nationalism shows the general characteristics of co-advance and retreat in the general trend, its four specific forms also show certain differences in the development direction. With the further setback of the globalization process, neo-nationalism will have a significant impact on the security of relevant countries, regions and even the world.

## Introduction

The world situation has undergone major changes since the 21st century. A striking phenomenon is that, ethnic and religious issues that used to be in a relative marginal or subordinate status have begun to move to the center of the political arena of various countries and even the world, standing as one of the central issues that some major countries in the world must face. New trends have emerged in the development of world ethnic issues correspondingly. On the one hand, traditional nationalism including state (religious) nationalism and local (ethnic) separatism still retains its vitality in the Third World countries. On the other hand, there have been various forms of nationalism in Western countries known for good governance, such as separatist nationalism and especially far-right nationalism and Christian evangelical nationalism. These interweaving nationalist thoughts and movements of the “Two Worlds” have brought great uncertainty to the world we live in. Most people feel insecure deep inside. How do we understand this phenomenon? While many considered this new phenomenon or trend of nationalism as a negative effect of globalization and neo-liberalism^1^, the Western media and academia termed such frequent nationalist phenomena as neo-nationalism^2^. Then, how do we view neo-nationalism (phenomenon)? What are the differences between neo-nationalism and traditional nationalism? Is neo-nationalism an episodic, transitional phenomenon, or a directional phenomenon that will change the political landscape of countries around the world from now on? This paper attempts to make a preliminary analysis.

## Methods

By using methods such as literature, norms, comparison, field observation, the paper makes an in-depth and systematic study into the formation process, origins, characteristics and trends of neo-nationalism in the 21 century, and the relations between neo-nationalism and identity politics as well. It points out that neo-nationalism will have a great impact on the security of related countries, regions and even the whole world as the globalization process suffers serious setbacks.

## Results and discussion

### Origins of neo-nationalism in the 21st century

The past few centuries have to some extent been characterized by traditional nationalism, ranging from nation-state building movement in Western European in the 17th − 19th century to the first two waves of nationalism in the 20th century. In fact, even the third wave of nationalism in the 20th century can generally be identified as traditional nationalism. These movements gave rise to a large number of independent nation-states, which, as the basic unit, formed and consolidated the world political pattern and international order.

In the 21st century, major changes have taken place in the world situation. Convergence and differentiation characterize the age of globalization––globalization of economic and democratic systems and polarization between the rich and the poor, as well as identity politics and alter-globalization. Waves of refugees have been caused by regional wars of invasion involving and led by major powers. Under the impact of these multiple factors, new nationalism that is clearly different from traditional nationalism has emerged worldwide, especially in Western countries. The so-called neo-nationalism has high degree of complexity and inherent conflict as it represents a confluence of many ideologies such as populism, anti-globalization, opposition to immigration, egoism, exclusionism, nativism, protectionism, isolationism, and faith politics. Its origins and connotations are much more complicated, whereas traditional nationalism can be simplified as “an idea and movement that puts self nation/ethic group in the highest place of the chain of political, economic, social, and cultural values chains”. There are more profound economic, political, social, cultural and psychological forces driving the rise of neo-nationalism. In Western countries, neo-nationalism is manifested in nativism against immigrants and other “foreigners”, anti-modern religious conservatism, and egoism with economic self-interest. In the Third World countries, neo-nationalism is reflected in faith and ancient nativism. That is to say, neo-nationalism is not a single form of nationalism distinct from traditional nationalism. It generalizes a specific type of nationalism and describes a range of new nationalism phenomena. In the former case, it includes far-right nationalism (Europe) and evangelical nationalism (the United States). In the latter case, it encompasses separatist nationalism featuring economic self-interest and religious nationalism (the Third World) that uses the faith politics to build and shape national identities.

### I the rise of far-right nationalism

Right-wing extremism has long been standing as a political faction. It achieved its apotheosis as fascism in a complete form of (statism) nationalism in the 1930 and 1940 s, but returned to the conservative (reactionary) political spectrum with the fall of extreme nationalism (fascism) after World War II. As a super ordinate concept of fascism, right-wing extremism was once completely denied politically and legally, so that over a long period of time, it has often been used by political parties as an important tool to criticize, smear and demonize each other (Camus et al. 2017).

In the 21st century, the issues of employment, security and identity are superposed amid all kinds of contradictions and conflicts. European far-right parties marginalized after World War II begin to assess the situation and reshape their image. They change ideas and adjust strategies in an attempt to waltz into the mainstream (The Economist 2018). In terms of political discourse, far-right parties covet the image of a normalized party fit to govern by eliminating the “politically incorrect” elements such as anti-Semitism and racism (Alduy 2016)^3^. In terms of campaign strategy, radical right-wing parties adopt a “people-friendly” strategy by drawing on the “positive energy” of traditional left-wing parties. The specific approach is to adopt individualized and targeted political strategies. For example, far-right parties take root at grassroots level in communities that encounter problems such as population outflow and social disintegration due to the transfer of traditional industries. They absorb a large number of unemployed young people and train them to become backbones, while highlighting job creation and community reconstruction in mobilization strategies. Far-right parties give these young people the direction of life and the sense of accomplishment, and leverage their influence to win the support of communities.

Far-right parties have changed the post-World War II embarrassing situation of surviving in the cracks by taking advantage of the negative effects in the process of globalization and regional integration, as well as the mistakes of traditional parties in national governance. However, without the basis of white racism or ethnic nationalism, the political storms they set off would be at most an extreme case of political party struggle, or a temporary unbalanced fragment of ideological struggle, and far from sort of nationalism. As said by Prof. Tamir Baron, without ethnic nationalism as its master concept, the radical right’s thinkers, political parties, and movements would lack a stable anchor to exert the profound impact of nationalism (Baron 2018).In fact, it is precisely the tortuous, implicit integration of the long-disgraced white racism (ethnic nationalism) into the political program that enables European far-right parties to stimulate every exclusive and dangerous nationalism in the extensive political mobilization process (Zhou 2016).

### II the rise of evangelical nationalism

Evangelical nationalism rose in the United States against a similar historical background to far-right nationalism, but has its own characteristics in the specific development path.

Evangelical nationalism originated from evangelical Christianity, which is an extremely important variable affecting American political, cultural and social life. In more than 200-year American history, evangelical Christianity has always served as an influential social force in the development of American politics. As early as the 18th and 19th centuries, evangelicals impacted and shaped the values of American society in the form of social movements such as the Great Awakening. Since the early 20th century, evangelicalism with relatively singular religious appeal has undergone a major political transformation to nationalism expressing the social and political appeals and aspirations of white groups. More precisely, it has gradually turned from religious conservatism to white racism. An obvious example is the 1954 Supreme Court case that ended racial segregation between black and white (public) schools in the United States, declaring the legal principle of “separate but equal” was unconstitutional. Afterwards, southern whites who were unwilling to be schooled with blacks fled public schools and established their own separate schools––private white schools. Then, the Moral Majority known for hard-line and conservative religious and moral values was founded. In 1980, the organization made known as a political force by helping Ronald Reagan take the helm at the White House. Since then, the predominantly white Christian faction that represented nearly a quarter of American population has become non-neglectable for any political forces. In 2016, evangelicalism was gradually evolved into evangelical nationalism while mobilizing support for Donald Trump’s presidential campaign (Zhou 2017) as a combined result of various factors (illegal immigration, the resistance of forgotten workers and farmers in globalization, the bold and even extreme liberalism of the Democratic administration, and the heavy legacy of racial issues, etc.).

Evangelical nationalism is a very subtle expression of white racism or white supremacism under the new historical conditions. Internally, it suppresses ethnic minorities, immigrants and women, as well as the homosexual groups by means of protecting “religious freedom”. Externally, it launches attacks on multiple fronts under the “America First” banner, such as politics, military and trade. Evangelical nationalism has a complete set of technical specifications and procedures for internal affairs, diplomacy and values.

The dramatic politicization of American evangelicalism is not simply a matter of church-state relations. Evangelical nationalism is a new-era variant of traditional white racism: Through the political assertion of Christian conservatism and a certain degree of national mobilization, American evangelical conservatism has developed from a religious conservative force into a complete form of nationalism. Evangelical nationalism gives anew expression to American statism (nationalism) in the 21st century^4^.

### III the resurgence of separatist nationalism

Separatist nationalism is a relatively common chronic problem facing countries around the world, with a history almost as long as nation-state. Since the inception of nation-states in Western Europe, the movement of trying to separate from the existing nation-states has never stopped^5^. Under the influence of the “one nation, one state” nation-state view in Western Europe, separatist nationalism has not only lingered in European and American developed countries, but also exerted significant impact on the vast number of developing countries^6^. Whether in developed countries in Europe and America or in developing countries, separatist nationalism generally aims to pursue territorial or political (cultural) autonomy/self-determination)^7^.

Separatist nationalism becomes active again in the 21st century. Apart from traditional motivations, this is clearly associated with a series of consequences brought about by globalization, such as the polarization between the rich and the poor, immigration (refugee) crisis, security and identity. Among the distinct features, separatist nationalism is driven by economic nationalism or egoism, noticeably in some regions of some developed countries such as Scotland in the United Kingdom, Catalonia in Spain, and Flemish Region in Belgium. In these countries, political and cultural identity is no longer an important issue (which is effectively alleviated through high degree of regional autonomy), and economic egoism and chauvinism are currently mainly used to mobilize the cohesive forces of separation.

In addition to economic motivation, the pursuit of self-security is also an important motivation of separatist nationalism in the 21st century. Besides, the post-1970s globalization of democracy gives legitimacy to separatist nationalism. Whether in developed countries or in the Third World countries, “democratization” and “partisanization” play a pivotal role in driving separatist movements.

### IV the proliferation of religious nationalism in the Third World

The proliferation of religious nationalism in the Third World is one of the important features of neo-nationalism in the 21st century. Most of the Third World countries are in the dual process of nation-state construction and democratization over a relatively short history of statehood. After entering the 21st century, inspired and exampled by far-right nationalism and evangelical nationalism especially in developed countries, religious nationalism has run rampant in some developing countries such as India (Zhou 2018a), Myanmar, Sri Lanka (Zhou 2015), Sudan, Pakistan, and Turkey. The governing parties or the military in these countries manipulate the “dominant religion” with the majority of the population to incite the religious sentiments of voters (congregants) in political elections and other major domestic issues, causing ethnic tension and political instability within the countries^8^.

It is worth noting that the Third World religious nationalism brings into play “ancient nativism” while using religious beliefs to create “others” for shaping national identity. The main driver of Hindu nationalism, the Bharatiya Janata Party (BJP), argue that Hindus have lived in India thousands of years ago: The “True Indian Nation” existed more than a thousand years before Muslims and other ethnic minorities “invaded and colonized” India. BJP’s mission is to restore and lead this ancient country towards” one race, one people, one language, one religion, one culture, one hope, one nation” (Misra 2000). Burmese Buddhist nationalists also see themselves as the “ancient masters” of this land, considering the Rohingya as outsiders or even “colonizers”. Obviously, the Third World religious nationalism is pushing Western European traditional nationalism towards polarization while drawing on its idea of “one nation, one people”.

### Characteristics of neo-nationalism

Compared with traditional nationalism, neo-nationalism has its own characteristics, including guiding ideology or values behind, field of occurrence, epochal character, propulsion mechanism, function, mode of communication, and influence.

In terms of guiding ideology or values behind, traditional nationalist movements mainly called for “freedom”, “equality” and “justice”, and integrated the interests, pride and patriotism of the nation with the pursuit of “justice” in a wider range. Neo-nationalists in the 21st century flagrantly put their own interests ahead of the interests of other groups and countries and openly agitate for self-interest as “America First “and “Hindu First”. They negate “political correctness” by questioning the existing value principles and political civilization achievements, and even plainly publicize meritocracy and the law of the jungle. Such throwback of values poses a severe threat to the existing international consensus and the world political order.

From the perspective of epochal character, traditional nationalist movements generally conformed to the requirements of the nation-state era, and gave rise to a series of modern nation-states that lay the foundation of a new international order. Neo-nationalism is adapted to anti-globalization or de-globalization. It attempts to restore the nation-state era by deconstructing the nation-state-based supranational governance (including regional integration and global governance), so it is essentially moving against the historical trend.

In terms of propulsion mechanism, traditional nationalism relies on elitism, while neo-nationalism resorts to populism. Traditional nationalism is more “political revolution” with the hue of “democratic revolution”. The nationalist elite have effectively designed, mobilized and driven the political revolution that changes the historical process and the world (regional) pattern. In contrast, neo-nationalism is more manifested as a kind of social movement. In this social movement that has swept across the world, the ordinary people influenced by right-wing forces have injected great impetus or even played a decisive role^9^.

From a functional point of view, neo-nationalism has turned negative overall due to retrogression in guiding ideology or values behind. It has been transformed into a tool for party (political) game from an anti-feudal, anti-theocratic political movement for national independence or autonomy in the era of traditional nationalism. In the separatist movement, nationalists especially of Western countries, largely strive for maximum economic interests instead of political and cultural self-determination. In the far-right movement, nationalism is simply an anti-institutional tool to pursue political power. In the evangelical movement, nationalism has become the most effective way for Republican Trump to seek and maintain state power. In the Third World religious movement, nationalism serves as the most important tool for the governing party to consolidate power and the opposition party to seize power^10^. Hence, the development of neo-nationalism not only undermines political integration and stability within the relevant countries, but also increasingly imperils the existing world order and international security.

On the mode of communication, neo-nationalism makes good use of the Internet and social media for face-to-face direct indoctrination to shape the values among the public, in addition to indirect indoctrination and dissemination via traditional media. Under this mode of communication, the masses act as the conscious disseminators at the forefront of neo-nationalism. It is precisely such mode that enables neo-nationalism to transcend the elite paradigm of traditional nationalism and amplify nationalist sentiment, identity and appeal to the extreme through the direct participation of the masses. Online forums, rallies and solidarity activities in various names have turned highly professional issues in specific fields for political negotiations into uncontrolled populist topics. In order to directly and timely inspire and respond to supporters, neo-nationalist leaders have almost invariably grown into the “masters” of high-tech national populism well using new media^11^.

From the perspective of influence, the traditional nationalist movement is often limited to specific countries and regions. However, neo-nationalism, especially far-right nationalism and evangelical nationalism, involves targets and activities beyond specific countries. Judging from the scope of penetration and influence, it has extended to become a major factor affecting regional and even global political order and political security.

Whereas the “problem countries” of traditional nationalism are mostly categorized into the Third World, the symptoms of neo-nationalism in the 21st century are seen in Western countries, including separatist nationalism, far-right nationalism, and evangelical nationalism at the moment. In fact, the more active presence of separatist nationalism and religious nationalism in the Third World in the 21st century is inseparable from the support, demonstration and motivation of various nationalisms in Western countries. Neo-nationalism is the product of irreconcilable contradictions between the nation-state theory of Western countries, state governance, and supranational governance (including regional integration and globalization) dominated by Western countries.

After the decolonization movement ended, the function or role of nationalism turned negative as a whole. In terms of discourse, nationalism became correspondingly an important tool for internal and external ideological struggles. For a long time, Western countries have always, intentionally or unintentionally, attached the label of “nationalism” to the Third World developing countries, in order to highlight the superiority of their “liberal democracies”. Ironically, however, Western countries themselves have fallen into the set pattern of neo-nationalism since the 21st century.

In addition, neo-nationalism in the 21st century has another distinct feature: various nationalisms inspire and compete with each other, producing a superimposed effect in terms of influence. In this sense, neo-nationalism in the 21st century can be deemed as a complex cascade of nationalism.

### Neo-nationalism and identity politics

Apart from characteristics described above, neo-nationalism is a kind of high-intensity identity politics. For a long time, identity politics has been, consciously or unconsciously, linked to the civil rights movement, the feminist movement, and the movement for equal rights of homosexual groups, which emerged in the United States in the 1960 and 1970 s. It is considered to be a movement against citizen politics, wherein people of a particular race, ethnicity, gender or religion politically align with the left wing in order to defend their own interests. However, a look into more distant history finds that the so-called identity politics is essentially a direct product of the nation-state movement underpinned by nationalism since the 16th-century Reformation. There would be no identity politics in the modern sense without the nation-state building movement of “demarcating boundary by ethnicity”. It is exactly the nation-state building behavior based on a specific identity^12^that foreruns identity politics. In other words, the primary driving force of identity politics comes from nationalism promoting the nation-state movement^13^.

The identity politics of neo-nationalism as compared with traditional nationalism has a sort of quasi-fundamentalist characteristics. It discards the core value principles of traditional nationalism and the basic etiquette of polite society (Rose 2019), and brings some dangerous and even crazy essential factors of nationalism to extremes in the new era (Luo 2015). Moreover, under the deceptive banner of protecting local people and returning to religion and traditional culture, the influence and destructiveness of neo-nationalism has been significantly enhanced as a large number of median voters who are neither nationalist nor racist are entangled into the camp.

Due to space limitations, a brief analysis of this assertion is hereby given through an example of evangelical nationalism.

After Trump was elected the President of the United States in 2016, Prof. Mark Lilla of Columbia University denounced Hillary for “losing that big vision and slipping into the rhetoric of diversity” in his article *The End of Identity Liberalism* published in *The New York Times*. He said that in the presidential election, Hillary called out explicitly to African-American, Latino, LGBT and women voters, leaving out the white working class and those with strong religious convictions. As a result, fully two-thirds of white voters without college degrees voted for Trump, as did over 80% of white evangelicals. “In recent years, American liberalism has slipped into a kind of moral panic about racial, gender and sexual identity that has distorted liberalism’s message and prevented it from becoming a unifying force capable of governing.“ Post-identity liberalism that emphasizes citizens and their duties and solidarity is needed to replace identity (liberal) politics, Lilla suggested (Lilla 2016).

Francis Fukuyama also classified the political process of socially marginalized groups demanding equality into identity politics in his two important research findings published in 2018 (Fukuyama 2018). The difference is that Fukuyama does not oppose identity politics as a whole, arguing that “there is nothing wrong with identity politics; it is a natural and inevitable response to injustice. It becomes wrong only when it is used to serve some specific purpose” (Abrams 2019). The remedy is to define “larger and more integrative national identities” (Fukuyama 2018) or use “belief-based national identities” to maximize the “common ground among citizens” (Abrams 2019), so as to realize the restriction and guidance of citizen politics on identity politics.

Clearly, both Lilla and Fukuyama attributed the rise of Trump’s evangelical nationalism with evangelicals to the identity politics of minorities and leftists. In their opinions, the identity politics of minorities or marginalized groups dominated by leftists or progressivists is the main reason behind the rise of Trump’s evangelical nationalism or white identity politics. Fukuyama further developed the theoretical framework of this theory of identity politics. He thought that the “desire for recognition” has played a huge driving role in the historical process of human development. It has two types: “Isothymia” is the demand to be respected on an equal basis with other people; and “megalothymia” is the desire to be recognized as superior (Abrams 2019). The former gives a driving force for liberal democracies, while the latter poses a huge threat to liberal democracies. Fukuyama went on to point out that in the contemporary world, identity politics has become the dominant concept explaining most international affairs. Unless such liberal democracies can work their way back to more universal understandings of human dignity, they will doom themselves—and the world—to continuing conflict (Fukuyama 2018).

It is worth noting that, in the view of many scholars including Fukuyama, whether it is the struggle of minorities or marginalized groups for equal rights, or the “resistance” of white mainstream groups in the pursuit of “equality” or even “superiority”, both can boil down to “identity politics”—even classifying the former as “primary identity politics” and the latter as “reactive identity politics”^14^.

There is no doubt that the nationalism or racism of white mainstream groups is a typical kind of identity politics. However, this is by no means “passive, negative consciousness awakening” and “self-protection” as some scholars said, but historically deeply ingrained self-consciousness. In fact, from a genetic perspective, the identity politics of white nationalism or racism inspired the identity politics of minorities and marginalized groups, not the other way around. Taking the United States as an example, the oppression of people of color, especially African-Americans and women, by white racism and patriarchy is observed throughout most of its history. In fact, it is only the long-term primary identity politics that sparked the resistance of minority groups, i.e., women, leading to the country-sweeping civil rights revolution in the 1960s. Stacey Abrams, an African-American female politician, believed that identity politics arose from the struggle of discriminated minorities and marginalized groups against mainstream groups. In this sense, identity politics is not so much created by leftists or minorities and marginalized groups as imposed by mainstream groups. Abrams viewed identity politics as “a tool for oppressed groups to fight for democratic rights and defend equal dignity” and held that “such struggle strengthens rather than threatens democracy” (Abrams 2019). In the American history, it is precisely because of the resistance of leftists, minorities and marginalized groups that drove forward the American democratic and liberal system and ultimately brought about “American belief” in the modern sense (Sides et al. 2019).

Identity politics is “a political form serving the demand of a specific identity, or taking a specific identity as a priority consideration and even political criterion, or driven by the expression of a specific identity itself” (Tan 2020). The resistance struggle of leftists, minorities and marginalized groups has certain characteristics of identity politics, but it is not the same as the identity politics of mainstream groups. The former strives for the same “equal treatment” as the majority group, and the integration-oriented political movement typically makes the country more just and inclusive. The latter pursues self-defined “equality” or even “superiority”, with an intention of isolation or even expulsion rather than assimilation^15^.In terms of ideology and values, the identity politics of minorities and marginalized groups under the left-wing leadership places focus on liberalism, egalitarianism and progressivism, while the identity politics of mainstream groups often falls back on nationalism and even racism^16^. This is evidently manifested in the United States, both in history and in reality^17^.

It is particularly noteworthy that evangelical nationalism has the characteristics of the “politics of faith” on top of the general characteristics of identity politics. Although the United States has long claimed to be a “secular liberal democracy”, Christianity has never stood aloof from the political process (Zhou 2014). For a long time after World War II, intimidated by the dominance of liberalism, Christian political conservative forces have lived under the wing of conservatism. In the 1970s, American religious right-wing movements attempted to make use of conservatism for a “fierce and protracted culture war”, but fail to succeed due to the “liberal” nature of American conservatism. Until the emergence of “Alternative Right” in 2010,the religious right has finally found strong allies that can break free from traditional conservatism while standing up to the progressive left.

Fully politicized evangelicals form the backbone of the religious right. The evangelical religious right has both the undertone of white nationalism and the strong features of the politics of faith^18^, which makes evangelical nationalism more exclusive.

A person’s identity is not a “natural” or spontaneous process, but “a result of political practice within specific boundaries” and “a functional manifestation of the modern state”. The persistent and comprehensive identity politicization of establishments or mainstream groups may lead to the further marginalization of minorities and vulnerable groups, and more seriously, the acute division and disorder of the whole society and even the collapse of the existing state. “When the existing state collapses, the relationship between ethnic groups will become similar to state relationship in an international system: Different ethnic groups will cannibalize each other for self-protection as the distinction between defensive and offensive ethnic mobilization disappears” (Ge 2016). By then, the state would regress to a “jungle society”. This is the most serious consequence of identity politics of establishments or mainstream groups.

Therefore, the high vigilance and reflection on the issue of identity politics should focus on the establishments or mainstream groups^19^. As to reason, only the identity politics of the establishments or majority groups is highly oriented to nationalism and even racism^20^, and much more destructive to a specific country and even the world political order than the identity politics of minorities and marginalized groups under the leadership of the left. This not only has been proved by the historical severe disasters caused by nationalism, but also can be observed in the current threats brought by nationalism, including the threat of evangelical nationalism to the order inside and outside the United States, the threat of far-right nationalism to the European order, and the huge threat of (the Third World) religious nationalism to the security of the local countries and regions.

### Trends of neo-nationalism

While neo-nationalism advances and retreats together in the general trend, its four specific orms vary to some degrees in the development direction.

Far-right nationalism and evangelical nationalism are a matter of great importance in the history of Western nation-states and in the history of so-called “democratic” and supranational governance^21^. Their presence suggests that Western liberal democracy cannot solve the issue of ethnicity (nationalism) once and for all. “History is far from being over” on the issue of nationalism^22^. The emergence of far-right nationalism and evangelical nationalism reflects the inherent flaws of Western nation-states, liberal democracies, and post-World War II dominating West-centered governance model marked by neo-liberal ideology and consumer and social culture.

Far-right nationalism and evangelical nationalism do not negate or question the existing national boundaries, but reinterpret nationalism under the stimulus of transnational movements such as globalization and regional integration. While recognizing the legitimacy of the existing national borders, they reckon national border security is subject to threat from heterogeneous inhabitants caused by the influx of immigrants and status quo of supranational governance (Eger et al. 2015).

The West is the birthplace of nationalism. Western countries are the forerunners of nation-states. For hundreds of years, they have paid a huge historical price to tame nationalism. Since the middle 20th century, the (democratic) liberalism of Western countries has basically addressed the problems of nationalism between countries and all kinds of nationalism within countries.

In the 21st century, the issues of interest (employment and welfare), security (terrorism) and (cultural and psychological) identity have been coupled in Western countries under the combined effect of the European debt crisis, financial crisis and refugee crisis. Such rare interactions have gravely shaken and undermined the political pattern and political ecology formed since World War II. In this sense, far-right nationalism and evangelical nationalism are not simply the products of liberal capitalism. They have a series of events as the necessary conditions, such as economic downturn, poor integration of immigrants, and refugee crisis. Therefore, it is foreseeable that with the gradual economic recovery of Western countries and the increasing integration of immigrants, especially the gradual alleviation of refugee crisis, far-right nationalism and evangelical nationalism will find it less possible to influence the political and social life of Western countries due to substantially weakened social foundation^23^.

In contrast, separatist nationalism is a long-term challenge to Western countries. In the 21st century, Western separatist nationalism shows a growing tendency to economically selfish and exclusionist nationalism. Nevertheless, economic resources and powers that can be transferred from the central government are increasingly limited. Given the democratized party-based systems and mechanisms of inter-ethnic politics (Zhou 2018b), it will be difficult for Western countries with deep-rooted nation-state view of “one nation, one state” to shake off the fetters of separatist nationalism in the foreseeable future.

Due to the complex demands in politics, culture and economy, separatist nationalism in the Third World countries is more intense and more challenging as compared with Western countries. In these countries, the pursuit of separation or independence is underpinned by political and cultural “self-determination” and backed by economic selfishness and exclusionism. In the dual process of nation-state construction and democratization, most of these countries suffer political instability and are subject to geopolitical intervention by major powers from time to time. Given this fact, separatist nationalism not only does more harm to the Third World countries than to the Western countries, but also is politically unsolvable^24^.

Among the various forms of nationalism, the most troublesome is the religious nationalism of the Third World countries. These countries dominated by religious people have learned nationalism from the West, but not the strategies and wisdom to deal with nationalism. In order to forge the social cohesion and unity of nation-states, the only resources they can utilize seem to be highly exclusive religious nationalism. However, religious nationalism divides different religious, ethnic and cultural groups in these countries while creating national identities. Moreover, under the influence of party politics and electoral politics, religious nationalist parties in some countries have frequently provoked incidents in surrounding areas that have led to tensions with neighboring countries and even endangered regional security.

It will take long for religious nationalism to recede in the Third World countries. Western countries as “respectful teachers” of nation-states are required to take the lead in addressing their problems of neo-nationalism, presupposing internal social revolution in these Third World countries, especially complete secularization.

## Conclusion

Historically, nationalism gave rise to nation-state in the modern sense and served as the prime force of the formation of modern world system. In this sense, it undoubtedly represents progressive force. However, needless to say, nationalism has caused unprecedented disasters in this historical process. Its huge destructive power has permeated a series of events, ranging from the large-scale religious wars and endless religious persecution since the Reformation in the 16th century to the two world wars in the first half of the 20th century that left hundreds of millions in an abyss of misery^25^, and the subsequent population cleansing and even genocide in many emerging countries are all permeated with the power of nationalism. In order to tame nationalism that brought huge disasters to mankind, Western countries took the lead in establishing a new system of international (internal) rules after World War II: (1) Formulating international norms to ward off nationalism between countries, such as the Charter of the United Nations, which stipulates that “all countries, big and small” are equal, and prohibits breaches of the sovereignty of other countries; (2) Establishing an international legal system of human rights to guard against nationalism, especially racism, within a country, which stipulates that “all human beings are born free and equal in dignity and rights” and that “each State Party to the present Convention undertakes to respect and to ensure to all individuals within its territory and subject to its jurisdiction the rights recognized in the present Convention, without distinction of any kind, such as race, color, sex, language, religion, political or other opinion, national or social origin, property, birth or other status”^26^. In order to effectively prevent the harm of various nationalisms at home and abroad, the United Nations, including its relevant agencies, has also established institutions, systems and mechanisms such as the Security Council, the International Criminal Court, and the inter-state accusation and the acceptance of individual complaints.

Following the establishment of the United Nations and the international human rights system, most western countries have entered the so-called era of liberal democracy. One of the main features is that they adopt a neutral position on ethnic issues, and organize the political and social life of the people in accordance with the civil rights and freedoms advocated by liberalism. Western scholars summarize this process as “the victory of liberalism over nationalism”. So far, Western countries seem to have completely shaken off the shackles of centuries-old nationalism and begun to move in all respects towards the era of the “civic state” longed for by liberalists.

From another perspective, the establishment of the idea and system of citizen-state also marks that post-Reformation centuries-old identity politics begins to end. Since then, there seems to be no such titles in the Western world as “Catholic state”, “Protestant state”, “Christian state”, “white state”, or “Anglo-Saxon state”. These countries would have a splendid title instead—“civic state”^27^ or “liberal democracy”. Fukuyama even vowed ambitiously after the end of the Cold War to bring this model of liberal democracy to the world.

In the 21st century, the “citizen nationalist state” that Western countries had painstakingly built up since World War II, began to shake and even turn around under the combined impact of multiple factors, including the negative effects of globalization, the waves of refugees caused by the direct invasion or intervention of major powers, and the gradual deviation of traditional liberal and left-wing forces^28^. In the United States, evangelical Christians, who make up nearly a quarter of the total population, have been drastically politicized, leading to an evangelical nationalist movement that directly affects American domestic and foreign policies. In highly secular Western Europe, anti-immigration (anti-refugee) and anti-EU sentiments become powerful tools to mobilize local people, especially white conservatives and rust-belt workers. Far-right nationalism has quickly risen and spread in Western European countries. At the same time, separatist movements still obsessed with “one nation, one state” show no sign of abatement, but have added wings of economic egoism and exclusionism.

While nationalism and even racism are on the rise in European and American countries, nationalism has grown in various names in some “Third World” countries that have always “learned from the West”. Of particular concern is religious nationalism in India and Myanmar, where state or national identity has not only required identification with a certain value or race, language and culture, but also required to be a devout Hindu or Buddhist. This fully demonstrates that religious nationalism is highly exclusive^29^.

“Nationalism is an infantile disease, the measles of mankind,“ said Albert Einstein (Viereck 1929). Robert Sapolsky was deeply upset with cognitive contributors to nationalism, arguing that “Humans seem not too far from chimpanzees in terms of group belonging: They are comfortable with the familiar and instinctively resist the unfamiliar. To overcome this, the opposite way is needed” (Lilla 2016).

At a critical period for human society development, an increasingly number of problems needs to be solved or alleviated through the concerted efforts of all countries in the world. However, we have seen such a scene: Western countries as the “leader” of liberal democracies are caught in the whirlpool of nationalism, and some Third World countries that have been developing well are also at the heels. It is even more worrying that the already precarious globalization and the enabling pattern of interrelated future of people all over the world are facing more severe challenges amid the global epidemic of COVID-19. Today in the 21st century, a major issue of global concern is whether modern nation-states represented by the West can free themselves from the troubles and ties of nationalism with huge destructive power and high historical inertia, that is, to overcome the “infantile disease” of nationalism and escape from the cognitive dilemma of “chimpanzees”.

## Data Availability

Not applicable.

## Footnotes

1 Research results in this area are numerous and will not be listed here.

2 In order to distinguish it from classical nationalism, Western academia and media collectively referred to far-right nationalism and evangelical nationalism as neo-nationalism, that is, new phenomena appearing in Western countries since the early 21st century. On a practical level, the United Kingdom’s withdrawal from the European Union and Trump’s election as the United States president are regarded as typical events of neo-nationalism. In this paper, the author gives an extended understanding of neo-nationalism to include both separatist nationalism and (the Third World) religious nationalism as they are obviously influenced or inspired by far-right nationalism and evangelical nationalism. It should be noted that although neo-nationalism has been observed as a phenomenon in early 21st century, but as an important theoretical and practical issue, it has attracted widespread attentions in just a few years. The issue was triggered by two events in 2016, i.e., the United Kingdom’s withdrawal from the European Union and Trump’s election as the United States President. While nationalism has become a central topic of politicians, academia and media in Europe and America in recent years, there are not yet systematic research findings on its definition, characteristics and trends. The existing studies are mostly limited to analyzing the phenomena and proposing coping strategies. Research results on neo-nationalism in Europe and America include Maureen A. Eger, Sarah Valdez. Neo-nationalism in Western Europe [J]. European Sociological Review, February 2015, 31 (1): 115–130; Tony Barber. A Renewed Nationalism Is Stalking Europe [N]. Financial Times, 2016-06-11; Jonah Goldberg. New Nationalism Amounts to Generic White Identity Politics [N]. Newsday, 2016-08-16; Bret Stephens. Trump’s Neo-nationalism [N]. The Wall Street Journal, 2016-11-21; Ryan Cooper. The Great Recession Gave Rise to Right-wing Populism [ N]. The Week, 2017-03-15; Suzanne Schneider. Nationalists of the world, Unite! [N]. Foreign Policy, 2018-01-08; Tamir Baron. The Radical Right and Nationalism [M] / / Jens Rydgren. The Oxford Handbook of the Radical Right. Oxford: Oxford University Press, 2018: 17–41; Sindre Bangstad. The New Nationalism and its Relationship to Islam, Diversity and Contestations over Nationalism in Europe and Canada [M]. London: Palgrave Macmillan UK, 2018; Emma Green. The Nationalists Take Washington [N]. The Atlantic, 2019-07-17; Austin Ruse. Social Conservatism and the New Nationalism [N]. Crisis Magazine, 2019-07-19. Research findings on neo-nationalism in the Third World Countries mainly include Sinan Baykent. Turkey: Toward a Third “Nationalist Front” Government [N]. Hürriyet Daily News, 2016-11-08; Ellen Barry, Suhasini Raj. Firebrand Hindu Cleric Ascends India’s Political Ladder [ N]. The New York Times, 2017-07-12; Tom Stevenson. Our Bodies Are Turkish, Our Souls Islamic! The Rise of Turkey’ s Ultra⁃nationalists [ N]. Middle East Eye, 2018-07-21; Omar Waraich. Pakistan’ s Populist Triumph [N]. The Atlantic, 2018-07-27; Bibhudatta Pradhan. The Man Stoking Nationalism in India Could Succeed Modi One Day [ N]. Bloomberg, 2019-11-28.

3 The translation of the French article is provided by Prof. Chenyan Zhuang of Minzu University of China.

4 American state nationalism has its own characteristics as American state-building differs sharply from classical Western European nation-state building in historical foundation and logical basis. For a long time, the United States has been regarded as a model of civic nationalism. Such civic nationalism is considered to be based on universalist value principles, at the core of which are classical liberalism and individualism advocated by the founding fathers of the United States. However, the country bears a quite profound imprint of racial nationalism over its history of more than 200 years, which is reflected not only in the long-term maintenance of black slavery and racial segregation, but also in the long-term restriction and discrimination against Asian Americans. After World War II, especially the abolition of institutionalized racial discrimination and segregation, racial nationalism became groundless in the United States. The “great mission” of expressing and declaring the nationalism of the United States historically fell into the various conservative thoughts and movements that emerged after the war. Finally, the Conservative Party stood out as the backbone of state nationalism. However, the mainstream conservative party has not been able to get rid of the inherent constraints or shackles of liberal values, so its declaration and practice of state nationalism has fallen short of the requirements and expectations of conservative people, especially evangelical Christians, Catholics, some Jews and white racists. Until Trump rose, conservative forces with evangelicals as the representative found their own political agents. The next day Trump was elected president, the right wing hailed “Trump’s nationalism” and “official recognition of the alt-right”, believing that “it’s a victory for white America”. With the full politicization of evangelicalism and the arrival of the Trump administration, the Republican Party achieved a major historical transformation, and the United States entered a period of evangelical nationalism that is a new form of American state nationalism.

5 Until today, there are still eight high-risk areas for separatism in Western Europe, such as Catalonia and Basque in Spain, Scotland in the United Kingdom, Flemish Region in Belgium, Corsica in France, Bavaria in Germany, and Veneto in Italy.

6 In Central and Eastern Europe, the Balkans and the Middle East, for example, the separatist movement poses a huge challenge to “inherently weak” or chaotic late-born nation-state order.

7 Some scholars argued that the majority ethnic separatist movements demand more preferential policies from the central government, rather than strive to create an independent state.

8 This is most typical in India. Modi-led Bharatiya Janata Party started out with Hindu nationalism and openly advocated “reshaping the Indian nation-state” with “Hindu characteristics” and values, which caused tensions and escalated conflicts between ethnic groups in the country. Following the revocation of Kashmir’s autonomous status and the announcement of the Citizenship Act, Hindu nationalists once again stirred up the people’s exclusion and xenophobia in the face of the common enemy of all mankind––the coronavirus. They blatantly slandered that “China created the coronavirus, and Muslims spread it to India”. It is foreseeable that with the continued deterioration of the epidemic, the internal and external harm of Hindu nationalism will further be multiplied. Please refer to Coronavirus: Islamophobia Concerns after India Mosque Outbreak [EB/OL]. (2020-04-03) [2010-07-11]. BBC News, https://www.bbc.com/news/world⁃asia⁃india-5214726.

9 A typical event is the election of Donald Trump as the United States President. Another example is the BJP’s landslide victory in 2019, after which Modi urged the world to recognize the “power of Indian democracy”. India with 600 million voters is known as “the largest democracy in the world”.

10 Eric Hobsbawm also thought that nationalism in its originating stage, both in Europe in the early 19th century or in Asian and African colonies in the 20th century, held high the national flag to expand and unite more groups of people, but later, it slipped to create separation, division and restriction among groups of people. Please refer to Luo Xin. A View of National History beyond Nationalism [J]. Beijing Cultural Review, 2015(4):42–49.

11 Examples include Le Pen (France), Trump (the United States) and Modi (India). Before surpassed by Trump, Modi held the Twitter record of the largest number of political tweets. Please also refer to Postel-Vinay, Karoline. How Neo-nationalism Went Global [EB/OL] (2017-03-14) [2020-09-03]. The conversation, https://theconveration.com/how-neo-nationalism-went-global-74095.

12 Broadly speaking, the anti-imperialist and anti-colonial national (state) independence movements that have occurred in the Asian and African continents since the 19th century also fall to this category.

13 In this sense, all forms of nationalist movements to date can be classified into identity politics.

14 There are also domestic scholars who regard the identity politics dominated by evangelical nationalism and far-right nationalism as “reactive identity politics”. They thought that white identity politics gets active again because of the vigorous development of the identity politics of “non-mainstream groups”, as well as the squeeze of identity discourse, the decline of political and economic status and the dilution of their sense of identity and existence felt by local and mainstream ethnic groups following the entry of a large number of immigrants. Please refer to Junyi Ma. Multi-ethnic Community Building and Governance––the Development and Impact of Identity Politics [J]. *Academics*, 2017 (2): 97–106 + 324.

15 The current white nationalists identified as evangelicals aim to build a white, single-race society dominated by evangelicals, going beyond the leading or dominant position in a multi-racial, multi-ethnic society. In order to realize this political fantasy, they have even proposed extreme programs of isolation and expulsion.

16 Kong Yuan said in his article *Identity Politics and World Order Evolution* (published in *International Economic Review*, No.4, 2019, pp.90–106) that before the rise of white identity politics, Western countries were essentially “a (racial) hierarchical social structure” and began to shift to “a social structure of equal confrontation” after the rise of white identity politics. The author agrees with Kong Yuan on “hierarchical social structure”, but does not agree to describe the identity politics dominated by systems or mainstream groups and the identity politics of leftists and minorities as “equal confrontation”. The author holds that after the rise of white identity politics, Western countries began to show signs of transition to racial (national) countries. It is this tendency (sign) that distinguishes the two possible outcomes of identity politics.

17 In order to change the “marginalized and disadvantaged” status long ignored by the establishment and the media, white mainstream groups represented by evangelical Christians are more inclined to regain the “lost paradise” by way of nationalism and even racism. It is not appropriate to conclude that white mainstream groups are all nationalist or even racist, but based on the available evidence, “it is reasonable to infer that an increasing number of white Americans no longer see racism as morally corrupt and they are willing to be tied with explicit white identity politics”. Please refer to Geoge Hawle. Making sense of the alt-right [M]. Columbia: Columbia University Press, 2017. Quoted from Zhou Lian. Alt-right and American politics [J]. Dushu, 2018(1):20–31.

18 Bart Bonikowski and Paul Dimaggio profoundly revealed the relationship between American nationalism, or national identity, and religious belief. They divided American nationalists by population, from low to high, into four categories: “disengaged nationalists”, “creedal nationalists”, “restrictive nationalists” and “ardent nationalists”. Among them, “ardent nationalists” accounted for the highest proportion (24%), which is about the same percentage as evangelicals (about 25%). Ponikoski and DiMaggio pointed out that although ardent nationalists refuse to admit that “true Americans” must be American-born Christians, they all view Jews, Muslims, agnostics, and naturalized citizens less than “truly Americans”. This consensus crosses party lines between Republicans and Democrats. Please refer to Bart Bonikowski, Paual Dimaggio. Varieties of American popular nationalism [J]. American sociological review, 2016,81(5):949–980.

19 Of course, for minorities, marginalized groups, liberals and left-wing camps, a major problem urgently to be solved is to incorporate the pursuit of equality into a broader universalist human rights framework, so that the tenet of “liberal political ideas accessible to people of different cultural backgrounds” will not be twisted into “adherence to a particular racial, ethnic or religious identity”.

20 Fukuyama regarded Trump as a representative of populist nationalism in the preface to the book *Identity: The Demand for Dignity and the Politics of Resentment*. This judgment actually implied his concerns that the identity politics of the establishments or majority groups will evolve into nationalism on a large scale. In the article *Against Identity Politics: New Tribalism and the Crisis of Democracy*, Fukuyama linked identity politics directly with the most primitive form of nationalism––new tribalism, pointing out that it seriously endangers modern democracy.

21 22 years ago, famous American commentator Fareed Zakaria expressed concerns about the rise of “illiberal democracy” in non-Western countries in his article published in *Foreign Affairs*. He held that democratic elections led to flourishing “authoritarianism and ultra-nationalism” in South America, North Africa, Middle East, the Balkans, South Asia and Southeast Asia. Zakaria said that “democracy has meant liberal democracy––a political system marked not only by free and fair elections, but also by the rule of law, a separation of powers, and the protection of basic liberties of speech, assembly, religion, and property,“ and termed “this latter bundle of freedoms” as “constitutional liberalism”. In Zakaria’s view, this model of democracy is castrated by extreme nationalism in non-Western countries. Obviously, he was overly optimistic about the resistance of such constitutional liberalism to nationalism. 22 years later, Zakaria commented as a current affairs presenter that “American democracy could be in peril”. Please refer to Fareed Zakaria. The rise of illiberal democracy [J]. Foreign Affairs, Nov/Dec 1997, 76(6); Fareed Zakaria fears American democracy could be in peril [EB/OL]. (2019-11-27) [2019-12-03] CNN, https://edition.cnn.com/videos/tv/2019/11/27/gps-1201-fareeds⁃take.cnn.

22 In recent years, Fukuyama has published a large number of papers on the topic of “the end of history”. In these works, Fukuyama strongly criticized the challenges brought by various nationalisms to “democratic liberalism” since the 21st century. Although he generally adhered to his previous basic views, he had to admit that it is premature to assert “the end of history”.

23 It is worth noting that with the large-scale coronavirus outbreak in Western countries, far-right nationalism and evangelical nationalism may gain new momentum. However, as a recent commentary published in the magazine *Foreign Affairs* stated, in the face of the coronavirus, all countries and peoples share one common goal of protecting this world. Before the whole world becomes secure, no nation, no country or no individual can enjoy security alone. Please refer to NGOZIOKONJOIWEALA. Finding a vaccine is only the first step [EB/OL]. (2020-04-30) [2020-06-27] Foreign Affairs, https://www.foreignaffairs.com/articles/world/2020-04-30/finding-vaccine-only-first⁃the step. In this sense, it is still difficult to draw a clear conclusion on how the coronavirus impact the direction of far-right nationalism and evangelical nationalism.

24 Hobsbawm once pointed out that “separatism cannot solve the problems faced by the late 20th century at all, because it can solve neither universal problems … nor even local problems. It will only make the situation worse, making ethnic problem more challenging.“ This argument is not outmoded today, whether for Western countries or for the Third World countries. Please refer to Eric Hobsbawn. *Nations and Nationalism* [M]. Li Jinmei translated. Shanghai: Shanghai People’s Publishing House, 2000: 203.

25 This includes the appalling Holocaust in Nazi Germany during World War II.

26 Article 1 of the Universal Declaration of Human Rights; Article 2 of the International Covenant on Civil and Political Rights. Please refer to the Project Group of Course in International Human Rights Law. Course in International Human Rights Law: Vol. 2 [M]. Beijing: Press of Chinese University of Politic Science and Law, 2002: 2,7.

27 It is also known as “citizen nationalist state”.

28 With the gradual gentrification of the traditional working class after World War II, liberals and leftists in Europe and the United States gradually shifted the target of “political liberation” to the disadvantaged and marginalized groups such as ethnic minorities, women, and homosexuals, without giving due attention and sympathy towards the white underclass beset with poverty and other social problems in the process of globalization. As a result, the white underclass has turned to “identity politics” as the mass base of evangelical and far-right nationalist movements.

29 After investigating Trump’s populist nationalism, Fukuyama said with concern that “unless the United States can curb this trend in its country, or it will continue to set a bad example for the rest of the world.“ Please refer to Francis Fukuyama. Identity: the demand for dignity and the politics of resentment [M]. New York: Farrar, Straus and Giroux, 2018: 133.
